# Diagnostic value of radiomics model based on gray-scale and contrast-enhanced ultrasound for inflammatory mass stage periductal mastitis/duct ectasia

**DOI:** 10.3389/fonc.2022.981106

**Published:** 2022-09-20

**Authors:** Yan Zheng, Lu Bai, Jie Sun, Lin Zhu, Renjun Huang, Shaofeng Duan, Fenglin Dong, Zaixiang Tang, Yonggang Li

**Affiliations:** ^1^ Department of Ultrasound, The First Affiliated Hospital of Soochow University, Suzhou, China; ^2^ Department of Biostatistics, School of Public Health, Medical College of Soochow University, Suzhou, China; ^3^ Jiangsu Key Laboratory of Preventive and Translational Medicine for Geriatric Diseases, Medical College of Soochow University, Suzhou, China; ^4^ Department of General Surgery, The First Affiliated Hospital of Soochow University, Suzhou, China; ^5^ Department of Radiology, The First Affiliated Hospital of Soochow University, Suzhou, China; ^6^ Precision Health Institution, GE Healthcare, Shanghai, China; ^7^ Institute of Medical Imaging, Soochow University, Suzhou, China; ^8^ National Clinical Research Center for Hematologic Diseases, The First Affiliated Hospital of Soochow University, Suzhou, China; ^9^ Suzhou Key Laboratory of Intelligent Medicine and Equipment, Soochow University, Suzhou, China

**Keywords:** breast cancer, mastitis, radiomics, ultrasound, contrast-enhanced ultrasound (CEUS)

## Abstract

**Objective:**

The present study aimed to investigate the clinical application value of the radiomics model based on gray-scale ultrasound (GSUS) and contrast-enhanced ultrasound (CEUS) images in the differentiation of inflammatory mass stage periductal mastitis/duct ectasia (IMSPDM/DE) and invasive ductal carcinoma (IDC).

**Methods:**

In this retrospective study, 254 patients (IMSPDM/DE: 129; IDC:125) were enrolled between January 2018 and December 2020 as a training cohort to develop the classification models. The radiomics features were extracted from the GSUS and CEUS images. The least absolute shrinkage and selection operator (LASSO) regression model was employed to select the corresponding features. Based on these selected features, logistic regression analysis was used to aid the construction of these three radiomics signatures (GSUS, CEUS and GSCEUS radiomics signature). In addition, 80 patients (IMSPDM/DE:40; IDC:40) were recruited between January 2021 and November 2021 and were used as the validation cohort. The best radiomics signature was selected. Based on the clinical parameters and the radiomics signature, a classification model was built. Finally, the classification model was assessed using nomogram and decision curve analyses.

**Results:**

Three radiomics signatures were able to differentiate IMSPDM/DE from IDC. The GSCEUS radiomics signature outperformed the other two radiomics signatures and the AUC, sensitivity, specificity, and accuracy were estimated to be 0.876, 0.756, 0.804, and 0.798 in the training cohort and 0.796, 0.675, 0.838 and 0.763 in the validation cohort, respectively. The lower patient age (p<0.001), higher neutrophil count (p<0.001), lack of pausimenia (p=0.023) and GSCEUS radiomics features (p<0.001) were independent risk factors of IMSPDM/DE. The classification model that included the clinical factors and the GSCEUS radiomics signature outperformed the GSCEUS radiomics signature alone (the AUC values of the training and validation cohorts were 0.962 and 0.891, respectively). The nomogram was applied to the validation cohort, reaching optimal discrimination, with an AUC value of 0.891, a sensitivity of 0.888, and a specificity of 0.750.

**Conclusions:**

The present study combined the clinical parameters with the GSCEUS radiomics signature and developed a nomogram. This GSCEUS radiomics-based classification model could be used to differentiate IMSPDM/DE from IDC in a non-invasive manner.

## Introduction

Periductal mastitis/duct ectasia (PDM/DE) is also known as periductal mastitis, mammary duct ectasia or plasma cell mastitis and is recognized as the most commonly encountered inflammation of the non-lactating breast (1, 2). The main pathological features of this condition are dilatation of the ducts, and fibrosis and inflammation around them (3). The main clinical manifestations of PDM/DE are breast pain, mass, nipple discharge, skin redness, and so on (4, 5). According to the pathological results and the clinical findings, it can be divided into ductal dilatation stage, inflammatory mass stage, abscess stage, and fistula stage (5, 6). The inflammatory mass stage of periductal mastitis/duct ectasia (IMSPDM/DE) often presents as a lump or mass in the breast and enlarged axillary lymph nodes in the absence of any signs of inflammation. On the conventional ultrasound examination, IMSPDM/DE lesions often present as irregular hypoechoic masses located in the subareolar area, with abundant blood supply and not circumscribed margins. According to the second edition of the American College of Radiology (ACR) Breast Imaging Reporting and Data System (BI-RADS) Ultrasound, these lesions are generally classified into category 4 lesions. They are very similar with regard to the clinical and radiological results of invasive ductal carcinoma (IDC), which is considered to be the most common histological type of breast cancer (7, 8). However, the treatment and prognosis of IMSPDM/DE are significantly different from those of IDC (9). The ability to accurately distinguish IMSPDM/DE from IDC preoperatively is therefore of great clinical significance for the diagnosis and management of patients with PDM/DE.

Gray-scale ultrasound (GSUS) is a conventional modality that is used to reveal morphologic characteristics of breast lesions. Contrast-enhanced ultrasound (CEUS) can be used to visualize the blood supply and microvascular distribution of breast lesions (10). Previous studies have shown that the accuracy of traditional ultrasound in diagnosing PDM/DE ranges from 79% to 82%, while the accuracy range of CEUS in diagnosing PDM/DE is 72-83% (11–13). Despite the significant progress made in the ultrasonographic techniques, the distinction of IMSPDM/DE from IDC is challenging when based on image findings. At present, the main approach of PDM/DE diagnosis is imaging-guided biopsy.

Radiomics is a data mining approach, which aims to extract high-dimensional data from clinical images so as to build diagnostic and prediction models to address relevant clinical questions (14, 15). The application of radiomics in breast lesions is frequently performed to distinguish malignant from benign breast lesions, classify breast cancer types, and predict the treatment response and recurrence risk, mostly by using MRI images (16, 17).

In this study, we developed radiomics models based on GSUS images, CEUS images and clinical data to differentiate IMSPDM/DE from IDC. It was expected that this approach would eventually reduce the number of invasive biopsies.

## Materials and methods

The present retrospective study was approved by the institutional ethics committee of the First Affiliated Hospital of Soochow University (FAHSU) and written informed consent was obtained from all patients.

### Subjects

The pathology and ultrasound databases in FAHSU were used to conduct a retrospective search and recruit IDC and PDM/DE patients between January 2018 and November 2021.

**Figure 1:**
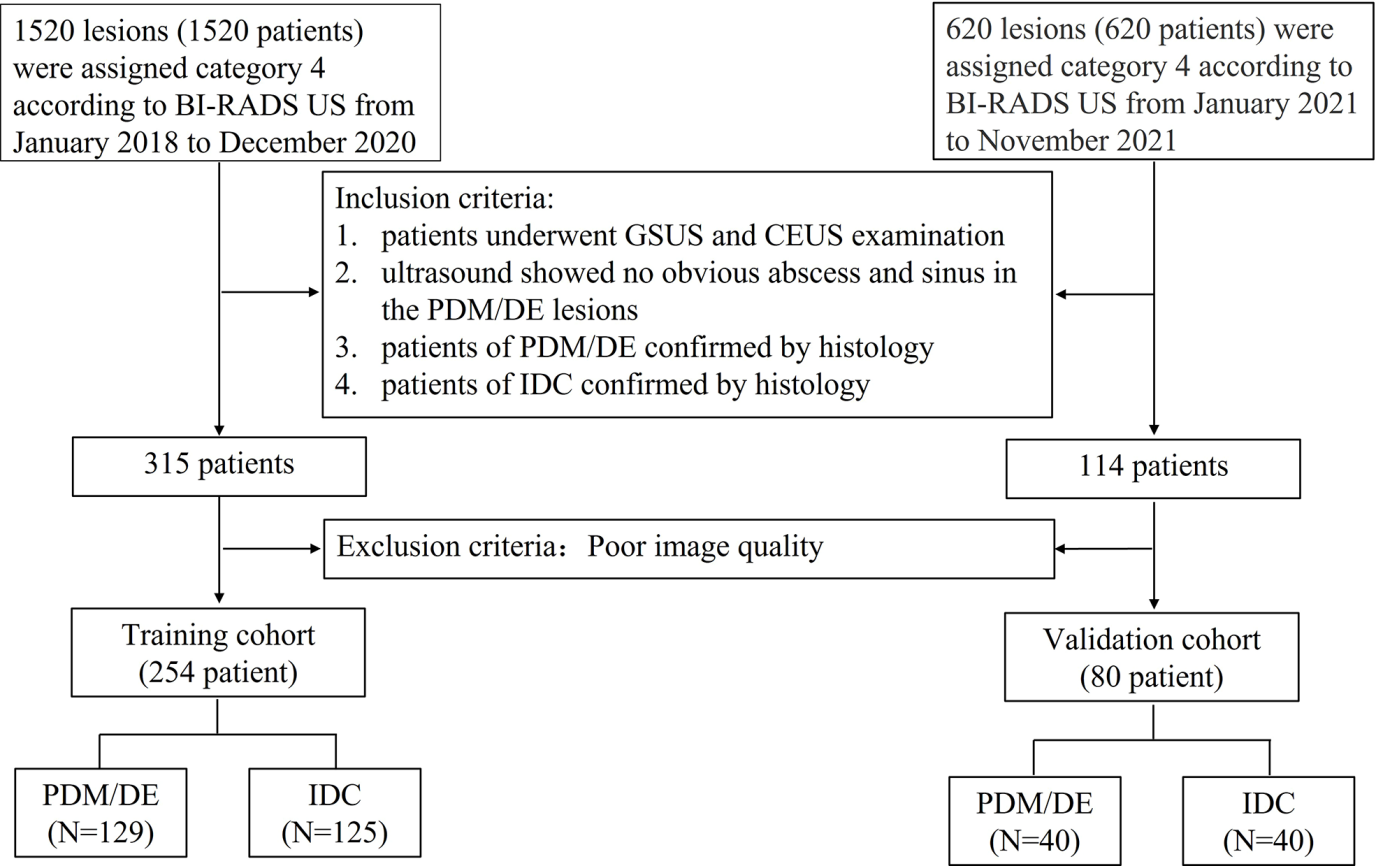
The flowchart of inclusion and exclusion of the study subjects. BI-RADS US Breast Imaging Reporting and Data System Ultrasound; GSUS gray scale ultrasound; CEUS contrast enhanced ultrasound; PDM/DE periductal mastitis/duct ectasia; IDC invasive ductal carcinoma.

The **inclusion criteria** were as follows: (1) Each lesion was assigned as category 4 according to the second edition of the ACR BI-RADS^®^ US Atlas; (2) all patients underwent GSUS and CEUS examination prior to biopsy; (3) the patients with PDM/DE were confirmed by histological analysis (gray scale ultrasound indicated no obvious abscess and sinus in the lesions); (3) the patients with IDC were confirmed by histological analysis; (4) the patients whose imaging quality of GSUS and CEUS met the requirement of analysis. The processes of inclusion and exclusion of study subjects are shown in **Figure 1**. The training cohort comprised patients (patients with PDM/DE or IDC) who were treated in FAHSU between January 2018 and December 2020 and the validation cohort comprised patients (patients with PDM/DE or IDC) who were treated in FAHSU between January 2021 and November 2021.

The following clinical parameters were used: Patient age, the maximum diameter of the lesions, the number of the lesions, the white blood cell (WBC) count, the number of monocytes, neutrophils, the presence of pausimenia, and the family history of breast cancer. All this information was derived from the medical records. The pathological specimens of each case were identified by experienced pathologists specializing on breast.

### Imaging acquisition and tumor segmentation

The ultrasound examinations were performed using one of the following ultrasound instruments: Mindray Resona7, LOGIQ E9, and MyLab™ ClassC equipped with high-frequency linear array probes (L14-6WU, L11-3U; ML6-15, 9L; and LA523 and LA522). To reduce microbubble destruction, low mechanical index (MI) values were applied (MI 0.02–0.07). The contrast agent used in this study was designated as SonoVue (Bracco SpA). The examinations were conducted by one of the three ultrasound practitioners with 10 years of experience in breast ultrasonography. The patients were placed in the supine or lateral position. The field of view was set to include the pectoralis muscle at the deepest aspect of the image. Gray-scale ultrasound scans were initially performed to identify the optimal scanning plane and save this image. Subsequently, CEUS examinations were performed, and their images were accompanied by the corresponding gray-scale images. The single frame corresponding to the moment of peak contrast perfusion in the lesion during CEUS was selected to represent the total process for radiomics analysis. The GSUS and CEUS images at the peak intensity were stored in Digital Imaging and Communications in Medicine (DICOM) format.

**Figure 2:**
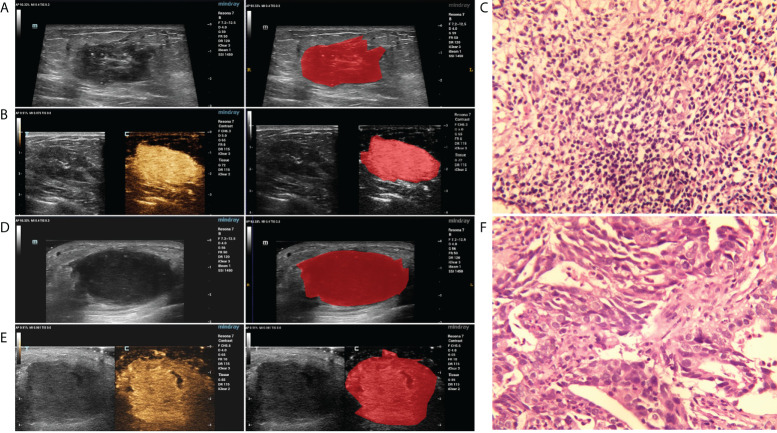
The representative results of the breast lesion segmentation. **(A, B)** The original GSUS, CEUS images and the segmentation of a 32-year-female patient confirmed with PDM/DE. **(C)** The sample (hematoxylin and eosin) with PDM/DE (400x). **(D, E)** The original GSUS, CEUS images and the segmentation of a 49-year-old female patient confirmed with IDC. **(F)** The sample (hematoxylin and eosin) with IDC (400x).

The region of interest (ROI) of the breast lesions was manually delineated using ITK-SNAP 3.8.0 software. Prior to segmentation, the CEUS and GSUS images were loaded into the software (MATLAB R2012a) and were converted from color maps to gray scales. Subsequently, the boundary of the breast lesion ROI delineation was performed by an ultrasound practitioner with experience in ultrasonography (>5 years), who was blinded to the clinical and histopathological data of the patients during the segmentation process. The representative results of the breast lesion ROI segmentation are displayed in **Figure 2**.

### Radiomics feature extraction, selection, and development of the radiomics signature

The radiomics features of the GSUS and CEUS images were extracted using Pyradiomics (version 2.1.1), which is an open-source Python package. The extracted features were classified into 6 categories as follows: Shape features, first-order statistical features, gray-level co-occurrence matrix (GLCM) features, gray-level run length matrix (GLRLM) features, gray-level size zone matrix (GLSZM) features and gray-level dependence matrix (GLDM) features. In addition, 8 filters, including wavelet-LLH, wavelet-LHL, wavelet-LHH, wavelet-HLL, wavelet-HLH, wavelet-HHL, wavelet-HHH, and wavelet-LLL, were applied to the original images, and the derived images were achieved for each patient. All classes of features, with the exception of shape, were computed on both the original and the derived images. Ultimately, 788 radiomics features were extracted for each ultrasound image.

Feature selection was carried out as follows: Firstly, the column containing “0” was deleted. Secondly, the Low Variance Filter method was used to remove the features which the variance was close to 0. If more than 95% of the data were the same in a feature, the feature was considered useless and was deleted. Thirdly, following data normalization (the mean value was subtracted from each feature and subsequently the values of each feature were divided by its standard deviation), the Select K Best method was used and the most important top K features were selected according to the P value (P<0.05). Subsequently, the top-ranking radiomics features of the GSUS and CEUS images were input to the least absolute shrinkage and selection operator (LASSO) classifier respectively, to select the most informative features.

Three radiomics signatures were then respectively developed using multivariate logistic regression (Stepwise regression) with the finally selected features of GSUS image and CEUS image. These included the GSUS radiomics signature, the CEUS radiomics signature, and the gray-scale combined contrast-enhanced ultrasound (GSCEUS) radiomics signature. The main indicators evaluating the performance of three radiomics signatures included AUC, sensitivity, and specificity. Subsequently, the best radiomics signature was selected.

### Development of radiomics-based classification model

The risk score for the radiomics signature of each patient (Radiomics_score) was calculated based on the β value of the selected radiomics features. To identify the significant clinical parameters, univariate analysis was performed on the following clinical parameters in the training set: Patient age, the maximal diameter of the lesions, the number of the lesions, the white blood cell (WBC) count, the number of monocytes, neutrophils, pausimenia and the family history of breast cancer. Subsequently, the clinical parameters with p<0.05 and the radiomics_score were included in the multivariate analysis to construct the radiomics-based classification model and differentiate IMSPDM/DE from IDC.

### Statistical analysis

The descriptive statistics were summarized as mean ± standard deviation (SD) or with the use of the 95% confidence interval (CI). The radiomics signature and the radiomics-based model were established by multivariate logistic regression analysis. The prediction performance of the radiomics signature and the radiomics-based model were assessed with the area under the receiver operating characteristic (ROC) curve analysis on the training and validation sets. The differences between various AUCs were compared using a Delong test. Sensitivity, specificity, and accuracy were also calculated. P values less than 0.05 indicated statistical significance. All statistical analyses were performed using the R statistical software (version 3.6.1).

## Results

### Patient profiles

A total of 334 patients including 169 patients (50.60%) with IMSPDM/DE and 165 patients (49.40%) with IDC were involved in the current study according to the inclusion and exclusion criteria. The subjects were all females with an age range of 22-89 years old (mean age, 41.90 years old). The training and test dataset consisted of 254 (129 IMSPDM/DE and 125 IDC) and 80 (40 IMSPDM/DE and 40 IDC) patients, respectively. The clinical characteristics of the training and validation sets are shown in **Table 1**. In the training cohort, the results indicated that the number of monocytes and the family history of breast cancer patients were not significantly different between the IMSPDM/DE and IDC groups (p>0.05). However, a significant difference was noted in the number of lesions in the IMSPDM/DE group compared with that of the IDC group (p<0.05). Statistically significant differences were noted in the patient age, maximal diameter of lesions, and pausimenia, as well as in the WBC and the neutrophil counts of the PDM/DE group compared with the differences noted in the IDC group (p<0.001).

**Table 1:** Clinical characteristics of patients on the training and validation cohorts.

Variables	Training cohort (n=254)			Validation cohort (n=80)		
	PDM/DE (n=129)	IDC (n=125)	*p* value	PDM/DE (n= 40)	IDC (n=40)	*p* value
Age (years)	33.49 ± 6.98	50.59 ± 13.37	< 0.001	33.95 ± 7.46	49.80 ± 11.19	< 0.001
Maximal diameter of lesions	36.18 ± 16.08	26.25 ± 12.69	< 0.001	31.88 ± 11.99	26.18 ± 11.29	0.0251
Number of lesions			0.019			0.009
Single	84 (65.12)	98 (78.40)		21 (52.50)	32 (80.00)	
Multiple (≥2)	45 (34.88)	27 (21.60)		19 (47.50)	8 (20.00)	
WBC (×10^9^/L)			<0.001			0.019
≤9.5	95 (73.64)	117 (93.60)		29 (72.50)	37 (92.50)	
>9.5	34 (26.36)	8 (6.40)		11 (27.50)	3 (7.50)	
Monocytes (×10^9^/L)			0.129			0.034
≤0.6	115 (89.15)	118 (94.40)		34 (85.00)	40 (100.00)	
>0.6	14 (10.85)	7 (5.60)		6 (15.00)	0 (0.00)	
Neutrophil (×10^9^/L)			<0.001			0.003
≤6.3	83 (64.34)	116 (92.80)		23 (57.50)	35 (87.50)	
>6.3	46 (35.66)	9 (7.20)		17 (42.50)	5 (12.50)	
Pausimenia			<0.001			<0.001
No	123 (95.35)	67 (53.60)		38 (95.00)	19 (47.50)	
Yes	6 (4.65)	58 (46.40)		2 (5.00)	21 (52.50)	
Family history			0.0655			0.4739
No	128 (99.22)	118 (94.40)		40 (100.00)	38 (95.00)	
Yes	1 (0.78)	7 (5.60)		0 (0.00)	2 (5.00)	

WBC, white blood cell.

### Feature selection and acquisition of radiomic signatures

In total, 1,576 imaging features were extracted from the GSUS and CEUS images of each patient (788 each).

**Figure 3:**
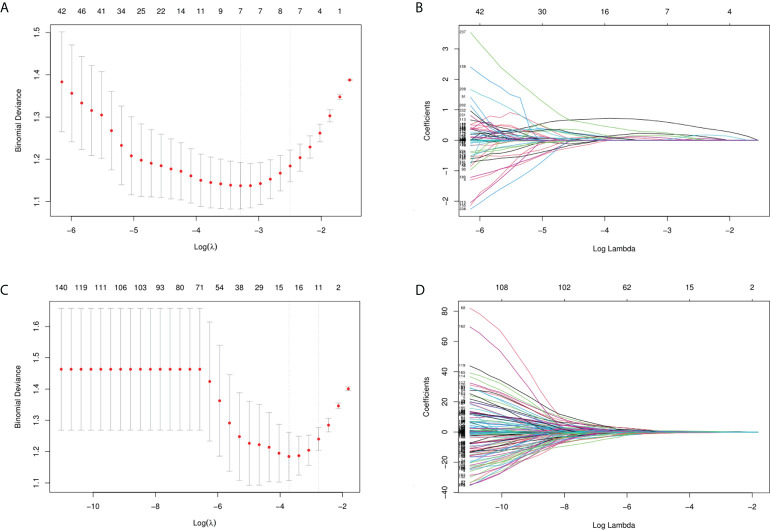
Feature selection. **(A, B)** Feature selection of GSUS images (λ= 0.0372, seven imaging features were selected); **(C, D)** Feature selection of CEUS images (λ= 0.0243, fifteen imaging features were selected).

In the training cohort, the features of which the column contained “0” and those with a variance close to 0 were excluded. Therefore, the number of GSUS and CEUS features was reduced to 365 and 372, respectively. Subsequently, the p value and the score of the Select K Best method were calculated, and the threshold for selecting the top-ranking radiomics features was p<0.05, leaving 260 and 236 features in the GSUS and CEUS, respectively. Thirdly, the LASSO algorithm was applied for subsequent feature reduction, 7 and 15 imaging features were selected respectively from the GSUS and CEUS images as potentially effective factors (**Figure 3**).

By using the Coarse-to-Fine Feature Selection strategy, 3 imaging features were selected for the construction of the GSUS radiomics signatures, and 6 imaging features were selected for the construction of the CEUS radiomics signatures. Finally, 6 imaging features were selected from the full feature set including 23 features of the above GSUS and CEUS features for the construction of the GSCEUS radiomics signature (**Table 2**).

**Table 2:** Radiomics features of three radiomics signatures.

GSUS radiomics signature	CEUS radiomics signature	GSCEUS radiomics signature
original_shape_MajorAxisLength	original_firstorder_InterquartileRange	wavelet.LLL_glcm_DifferenceEntropy(GSUS feature)
original_firstorder_Variance	wavelet.LLH_firstorder_Kurtosis	original_shape_MajorAxisLength (CEUS feature)
wavelet.LLL_glcm_DifferenceEntropy	wavelet.LLH_glrlm_LongRunEmphasis	original_firstorder_Energy(CEUS feature)
	wavelet.HLL_firstorder_Energy	original_firstorder_InterquartileRange(CEUS feature)
	wavelet.HLL_firstorder_Kurtosis	wavelet.HLL_firstorder_Kurtosis (CEUS feature)
	wavelet.LLL_glcm_DifferenceAverage	wavelet.LLL_glcm_DifferenceAverage(CEUS feature)

**Figure 4:**
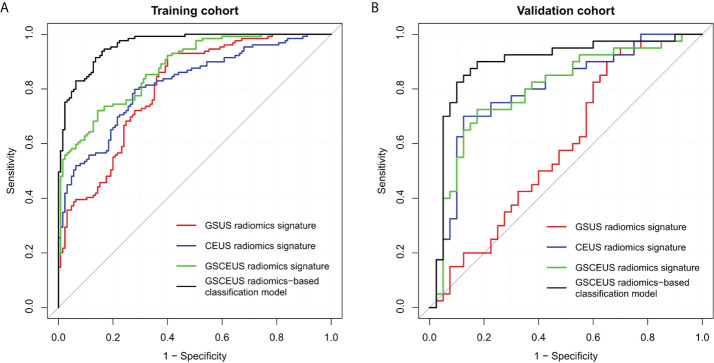
Receiver operating characteristic (ROC) curves of three radiomics signatures, and radiomics-based classification model to differentiate PDM/DE from IDC. **(A)** Four methods in the training cohort; **(B)** Four methods in the validation cohort.

The performance of the three radiomics signatures is summarized in **Table 3** and the ROC curves of the models are depicted in **Figure 4**. No significant differences were noted between the GSUS radiomics signature and the CEUS radiomics signature in the training cohort (AUCs 0.804 vs. 0.818; p=0.682). However, the CEUS radiomics signature performed better than the GSUS radiomics signature in the validation cohort (AUCs 0.797 vs. 0.590; p=0.003). The GSCEUS radiomics signature achieved optimal diagnostic efficacy for differentiating between PDM/DE and IDC compared with the GSUS radiomics signature in both the training (AUCs 0.876 vs. 0.804; p=0.001) and the validation cohorts (AUCs 0.796 vs. 0.590; p<0.001). Moreover, the GSCEUS radiomics signature performed better than the CEUS radiomics signature in the training cohort (AUCs 0.876 vs. 0.818; p=0.003). Therefore, the GSCEUS radiomics signature was used for further analysis.

**Table 3:** Predictive efficacy of radiomics signature and the radiomics-based model.

Different models	Training cohort (n=254)				Validation cohort (n=80)			
	Sensitivity	Specificity	Accuracy	AUC	Sensitivity	Specificity	Accuracy	AUC
GSUS radiomics signature	0.861 ± 0.109	0.640 ± 0.120	0.766 ± 0.046	0.804 ± 0.053	0.913 ± 0.087	0.312 ± 0.138	0.613 ± 0.075	0.590 ± 0.127
CEUS radiomics signature	0.698 ± 0.163	0.808 ± 0.152	0.766 ± 0.049	0.818 ± 0.051	0.775 ± 0.125	0.663 ± 0.138	0.725 ± 0.100	0.797 ± 0.101
GSCEUS radiomics signature	0.756 ± 0.198	0.804 ± 0.188	0.798 ± 0.046	0.876 ± 0.040	0.675 ± 0.150	0.838 ± 0.113	0.763 ± 0.088	0.796 ± 0.102
GSCEUS radiomics-based model	0.891 ± 0.093	0.884 ± 0.092	0.898 ± 0.032	0.962 ± 0.019	0.888 ± 0.088	0.750 ± 0.125	0.819 ± 0.081	0.891 ± 0.081

Six features of the GSCEUS radiomics signature were applied in the risk score calculation. The following radiomics_score calculation formula was used:

Radiomics_score = 3.117(wavelet.LLL_glcm_DifferenceEntropy) (GSUS feature)

+0.006(original_shape_MajorAxisLength) (CEUS feature)

+1.0e-09(original_firstorder_Energy) (CEUS feature)

+0.057(original_firstorder_InterquartileRange) (CEUS feature)

+ 0.081(wavelet.HLL_firstorder_Kurtosis) (CEUS feature)

-0.135(wavelet.LLL_glcm_DifferenceAverage) (CEUS feature)

The clinical parameters with p<0.05 in **Table 1** and the radiomics_score were included in the multivariate analysis (**Table 4**). A radiomics-based classification model was built by incorporating the parameters patient age, neutrophil, pausimenia and the radiomics_score. The performance of the classification model is summarized in **Table 3**, and the ROC curves of this model are depicted in **Figure 4**. In the training and validation cohorts, the sensitivity, accuracy and AUC values of the classification model were improved when the clinical parameters were added to the GSCEUS radiomics signature.

**Table 4:** Multivariate logistic regression analyses.

Characteristics	Multivariate analysis		
	OR	95%CI	p value
Patient’s age	0.81	0.74, 0.87	<0.001
Neutrophil	18.9	3.95, 123	<0.001
Pausimenia	0.11	0.02, 0.72	0.023
Radiomics_score	2.96	2.18, 4.30	<0.001

OR odds ratio; CI confidence interval.

**Figure 5:**
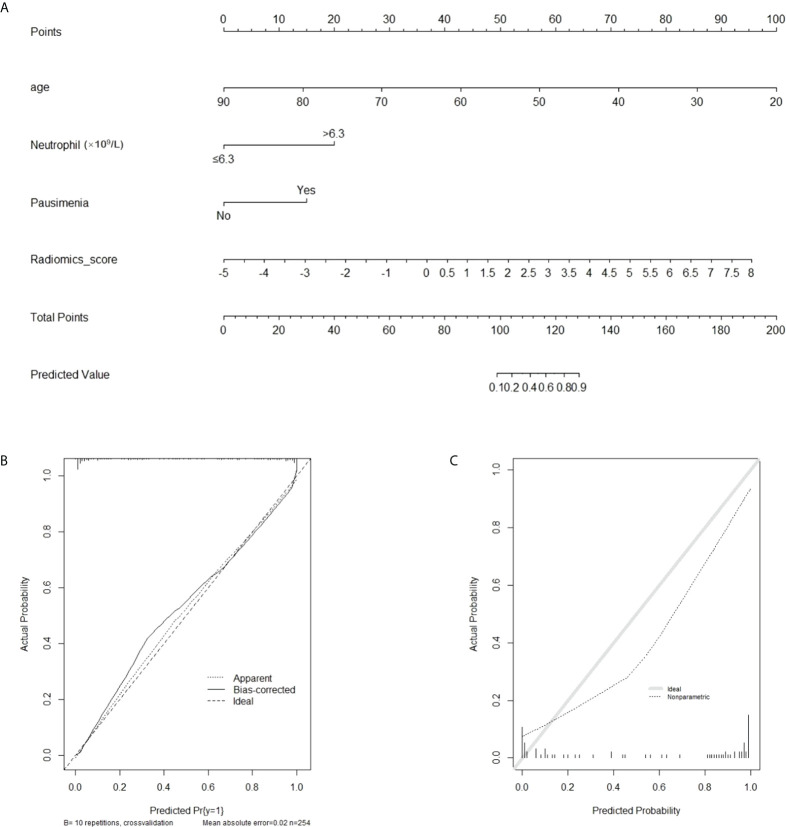
The radiomics-based nomogram for differentiating PDM/DE from IDC. **(A)** The radiomics-based nomogram developed with the training cohort included patient’s age and radiomics signatures. **(B, C)** Calibration curves of the radiomics-based classification model in the training **(B)** and validation **(C)** cohorts.

A nomogram of PDM/DE diagnosis was constructed using the aforementioned independent risk factors shown in **Figure 5**. The 10-fold cross-validation method was used for external validation of the generated model. The calibration curves of the validation cohort were plotted graphically and demonstrated optimal agreement between the diagnostic accuracy estimation by the nomogram and the histopathological confirmation (calibration intercept: -0.2685; calibration slope: 0.4888).

**Figure 6:**
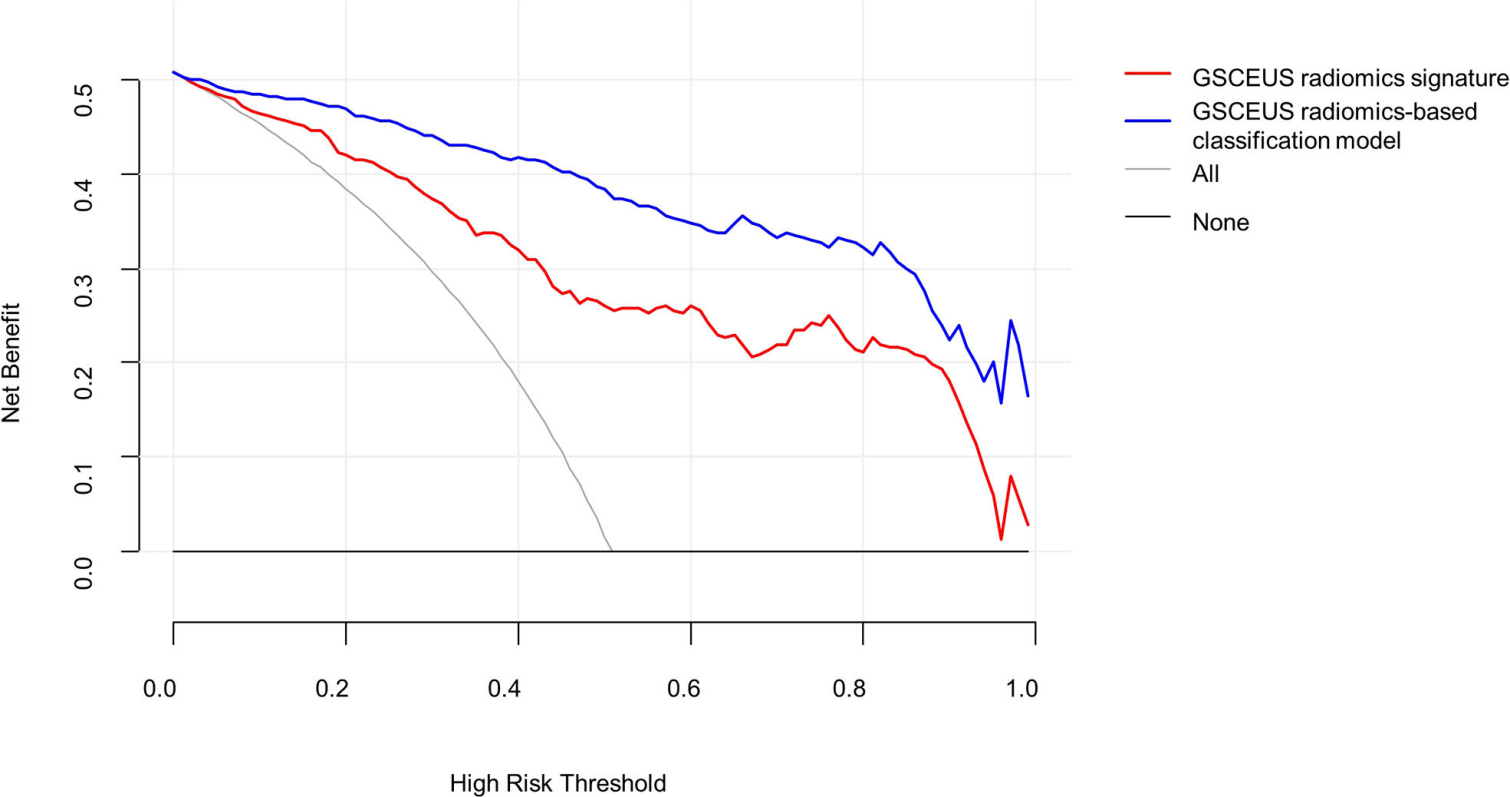
Decision curve analysis (DCA) derived from the validation group. The y-axis measures the net benefit. The net benefit is determined by calculating the difference between the expected benefit and the expected harm associated with each proposed model. If the threshold probability was more than 2%, using the nomogram to predict IMSPDM/DE added more benefit than either the treat-all scheme (grey line) or the treat-none scheme (dark black line).

A decision curve analysis was used to assess the clinical usefulness of the classification model and the GSCEUS radiomics signature in the validation cohort (**Figure 6**). If the threshold probability was more than 2%, the use of the classification model for the diagnosis of IMSPDM/DE added higher diagnostic value than either the treat-all scheme (assuming that all lesions were IMSPDM/DE) or the treat-none scheme (assuming that all lesions were IDC). In addition, the use of the classification model for the diagnosis of IMSPDM/DE added higher diagnostic value than that of the GSCEUS radiomics signature.

## Discussion

The experienced ultrasound doctor could distinguish IMSPDM/DE from IDC by lesion morphology, echo intensity, calcification, the blood supply of the lesions and the CEUS features. However, the ultrasound appearance of IMSPDM/DE could exhibit bewildering variation, suggesting that an inexperienced practitioner may experience difficulties in classifying between IMSPDM/DE and IDC (18–20). In the present study, the GSUS and CEUS-based radiomics features were used to differentiate IMSPDM/DE from IDC. The results revealed that the ultrasound-based radiomics features were able to distinguish IMSPDM/DE from IDC, whereas the GSCEUS radiomics features outperformed other radiomics features. Furthermore, a classification model was developed and validated. This model incorporated clinical parameters with GSCEUS radiomics features and exhibited high accuracy in differentiating IMSPDM/DE from IDC. The calibration curve indicated that the predicted and actual probability of IMSPDM/DE were in good agreement.

In order to construct a reliable radiomics model, the radiomics features were extracted from the GSUS and CEUS images, respectively. The key processes of the radiomics model construction included feature extraction, feature selection, and model construction (21, 22). In the feature extraction process, an open-source Python package (Pyradiomics) was used and 788 features were extracted on each image. In the feature selection process, the Low Variance Filter method, select K Best method and LASSO method were employed to avoid the curse of dimensionality. Subsequently, three radiomics signatures were constructed based on the radiomics features extracted from the GSUS and CEUS images. In both the training and validation cohorts, the GSCEUS radiomics signature demonstrated the highest diagnostic accuracy compared with those of the GSUS and CEUS radiomics signatures (training cohort:0.798 vs. 0.766, 0.766; validation cohort:0.763 vs. 0.725, 0.613). The AUC value of the GSCEUS radiomics signature was estimated to be 0.876 in the training cohort, which was significantly higher than that of the other two methods (AUC value: 0.818, 0.804). The CEUS radiomics signature indicated a similar AUC with that of the GSCEUS radiomics signature in the validation cohort (AUC value: 0.797 vs. 0.796), whereas the specificity and accuracy of the GSCEUS radiomics signature were better than those of the CEUS radiomics signature (specificity:0.838 vs. 0.663; accuracy: 0.763 vs. 0.725). The GSUS radiomics signature indicated a similar AUC with that of the CEUS radiomics signature in the training cohort (AUC value: 0.804 vs. 0.818), whereas the AUC and accuracy of the GSUS radiomics signature were significantly lower than those of the CEUS radiomics signature in the validation cohort (AUC value:0.590 vs. 0.797; accuracy: 0.613 vs. 0.725). In present study, the AUC value and accuracy of the GSUS radiomics signature in the validation cohort were significantly lower than those in the training cohort (AUC value: 0.590 vs. 0.804; accuracy: 0.613 vs. 0.766), which may be caused by different ultrasound systems. The training cohort contains images of three ultrasound systems, while the validation cohort contains images of only one of the above ultrasound systems. These results indicated that the radiomics features of the multimodal ultrasound imaging could make a critical contribution in improving the accuracy of the method. The GSCEUS radiomics signature with the best performance included 6 radiomics features in total, of which one was from the GSUS images and five were from the CEUS images. Half of the selected radiomics features in our study were wavelet-based features, which could presumably redisplay hidden tumor characteristics behind the speckle and increase the discriminative ability (23).

Certain clinical parameters related to mastitis and breast cancer were included in the present study, such as patient age, lesion size, WBC count, monocyte count, neutrophil count, pausimenia, and family history of breast cancer. Univariate analysis indicated that PDM/DE was more common in younger women and those without pausimenia (mean age: 33.49 ± 6.98), while IDC was more common in older women and in women with pausimenia (mean age: 50.59 ± 13.37). These findings are consistent with those of previous studies (8, 9, 18). Compared with IDC, the lesion of IMSPDM/DE was often larger and multiple. Although the etiology of PDM/DE remains unclear (5), it is a benign inflammatory disease, and its nature is different from IDC. Therefore, blood cell analysis are used as indicators of systemic inflammation and can potentially distinguish PDM/DE from IDC. The data of the present study demonstrated that 26.56% of IMSPDM/DE patients had increased WBC count, whereas 35.65% of IMSPDM/DE patients demonstrated increased neutrophil count, which was statistically significant compared with that of the IDC patients. No significant differences were noted in the monocyte count between the IMSPDM/DE and IDC patients. Neutrophils and WBCs are non-specific inflammatory markers, which can be used to indicate active bacterial infection. Breast cancer lesions rarely present with active bacterial infections. Therefore, the WBC and neutrophil counts may be used to differentiate PDM/DE from IDC. Family history is a major risk factor for breast cancer; approximately 5-10% of cases with breast cancer are associated with a family history of this disease (24, 25). However, the present study indicated no significant differences in the family history between the IDC and PDM/DE groups, which may be caused by the small number of patients included.

Multivariate analysis indicated that the variables patient age, neutrophil count, absence of pausimenia, and GSCEUS radiomics features were independent factors that could be used to differentiate IMSPDM/DE from IDC. Subsequently, a classification model was developed that could incorporate significant clinical parameters with the GSCEUS radiomics features. The accuracy of this model in the training and validation cohorts was 0.898 and 0.819, respectively, higher than 72%-79% reported in previous studies (11–13). The model was successfully validated and the data indicated that it could significantly improve the values of AUC in both the training and validation cohorts. The nomogram was primarily used to improve personalized diagnostics. The results of the present study suggest that the radiomics classification model, which was based on the GSCEUS images, could be used in a non-invasive manner to distinguish between IMSPDM/DE and IDC thus avoiding unnecessary biopsies; this application may facilitate the personalized treatment planning for these patients.

The present study contains certain limitations. Firstly, this was a single-center retrospective study and further multi-center studies with external authentication protocols should be conducted. Secondly, different ultrasound systems and scanning parameters may influence the generality of the results.

In conclusion, the current study developed and validated the radiomics classification model based on the GSCEUS radiomics signature, patient age, neutrophil count and absence of pausimenia. This model successfully distinguished IMSPDM/DE from IDC, and has the potential to avoid unnecessary biopsies.

## Data availability statement

The original contributions presented in the study are included in the article. Further inquiries can be directed to the corresponding authors.

## Ethics statement

The studies involving human participants were reviewed and approved by the Ethics Committee of the First Affiliated Hospital of Soochow University. Individual consent for this retrospective analysis was waived.

## Author contributions

YZ, LB, and JS contributed equally to this study. Conception and design of the study: FD, ZT and YL. Ultrasound and CEUS data acquisition: YZ, FD. Clinical and pathological data collection: JS, RH, and LZ. Analysis and interpretation of data: LB, YZ, JS, and SD. Drafting the manuscript: YZ. Discussion and manuscript revision: YZ, JS, LB, FD, ZT, and YL. All authors contributed to the article and approved the submitted version.

## Funding

This work was supported by the program for Gusu Medical Talent of Suzhou City (GSWS2020009); the Translational Research Grant of NCRCH (2020WSB06); the National Natural Science Foundation of China (No. 81671743, No. 81773541); the Suzhou Clinical Key Diseases Diagnosis and Treatment Technology Special Project (LCZX202104).

## Conflict of interest

Author SD was employed by GE Healthcare.

The remaining authors declare that the research was conducted in the absence of any commercial or financial relationships that could be construed as a potential conflict of interest.

## Publisher’s note

All claims expressed in this article are solely those of the authors and do not necessarily represent those of their affiliated organizations, or those of the publisher, the editors and the reviewers. Any product that may be evaluated in this article, or claim that may be made by its manufacturer, is not guaranteed or endorsed by the publisher.
